# Feasibility and safety of low-flow extracorporeal carbon dioxide removal to facilitate ultra-protective ventilation in patients with moderate acute respiratory distress sindrome

**DOI:** 10.1186/s13054-016-1211-y

**Published:** 2016-02-10

**Authors:** Vito Fanelli, Marco V. Ranieri, Jordi Mancebo, Onnen Moerer, Michael Quintel, Scott Morley, Indalecio Moran, Francisco Parrilla, Andrea Costamagna, Marco Gaudiosi, Alain Combes

**Affiliations:** 1 Department of Anesthesia and Critical Care - AOU Città della Salute e della Scienza di Torino, University of Turin, Corso Dogliotti 14, 10126 Torino, Italy; 2 Dipartimento di Anestesia e Rianimazione, Ospedale Policlinico Umberto I, Sapienza Università di Roma, Rome, Italy; 3 Servei de Medicina Intensiva, Hospital de Sant Pau, Barcelona, Spain; 4 Department of Anesthesiology, Emergency and Intensive Care Medicine, University Medical Center Göttingen, Göttingen, Germany; 5 ALung Technologies, Pittsburgh, USA; 6 Service de Réanimation Médicale, iCAN, Institute of Cardiometabolism and Nutrition, Hôpital de la Pitié-Salpêtrière, Assistance Publique-Hôpitaux de Paris, Paris, France

**Keywords:** Acute respiratory distress syndrome, Protective mechanical ventilation, Extracorporeal carbon dioxide removal, Extracorporeal membrane oxygenation, Positive end-expiratory pressure, Driving pressure, Ventilator-induced lung injury

## Abstract

**Background:**

Mechanical ventilation with a tidal volume (V_T_) of 6 mL/kg/predicted body weight (PBW), to maintain plateau pressure (P_plat_) lower than 30 cmH_2_O, does not completely avoid the risk of ventilator induced lung injury (VILI). The aim of this study was to evaluate safety and feasibility of a ventilation strategy consisting of very low V_T_ combined with extracorporeal carbon dioxide removal (ECCO_2_R).

**Methods:**

In fifteen patients with moderate ARDS, V_T_ was reduced from baseline to 4 mL/kg PBW while PEEP was increased to target a plateau pressure – (P_plat_) between 23 and 25 cmH_2_O. Low-flow ECCO_2_R was initiated when respiratory acidosis developed (pH < 7.25, PaCO_2_ > 60 mmHg). Ventilation parameters (V_T_, respiratory rate, PEEP), respiratory compliance (C_RS_), driving pressure (DeltaP = V_T_/C_RS_), arterial blood gases, and ECCO_2_R system operational characteristics were collected during the period of ultra-protective ventilation. Patients were weaned from ECCO_2_R when PaO_2_/FiO_2_ was higher than 200 and could tolerate conventional ventilation settings. Complications, mortality at day 28, need for prone positioning and extracorporeal membrane oxygenation, and data on weaning from both MV and ECCO_2_R were also collected.

**Results:**

During the 2 h run in phase, V_T_ reduction from baseline (6.2 mL/kg PBW) to approximately 4 mL/kg PBW caused respiratory acidosis (pH < 7.25) in all fifteen patients. At steady state, ECCO_2_R with an average blood flow of 435 mL/min and sweep gas flow of 10 L/min was effective at correcting pH and PaCO_2_ to within 10 % of baseline values. PEEP values tended to increase at V_T_ of 4 mL/kg from 12.2 to 14.5 cmH_2_O, but this change was not statistically significant. Driving pressure was significantly reduced during the first two days compared to baseline (from 13.9 to 11.6 cmH_2_O; p < 0.05) and there were no significant differences in the values of respiratory system compliance. Rescue therapies for life threatening hypoxemia such as prone position and ECMO were necessary in four and two patients, respectively. Only two study-related adverse events were observed (intravascular hemolysis and femoral catheter kinking).

**Conclusions:**

The low-flow ECCO_2_R system safely facilitates a low volume, low pressure ultra-protective mechanical ventilation strategy in patients with moderate ARDS.

## Background

Over-distention of the normally aerated lung and/or opening and closing of collapsed alveoli may worsen pulmonary damage in patients with acute respiratory distress syndrome (ARDS). Current guidelines for ARDS recommend a protective ventilation strategy based on limitation of tidal volume (V_T_) to 6 mL/kg predicted body weight and plateau pressure (P_plat_) to 30 cmH_2_O, an approach that has been shown in a randomized clinical trial to reduce mortality by 9 % [1]. However, recent studies have shown that ARDS patients who are ventilated according to the ARDS Network (ARDSnet) protective ventilatory strategy may still be exposed to forces that can induce lung injury [2–5], thus challenging current recommendations on how to minimize the risk of ventilator-induced lung injury (VILI) [3]. Moreover, Hager and coworkers [6] showed that mortality decreases as P_plat_ is reduced. However, as this relationship appears to be linear [6], several authors have postulated that an ultra-protective ventilation strategy based on further reduction in V_T_ from 6–4 mL/kg and P_plat_ from 30–25 cmH_2_O may improve outcomes [3]. Such tidal volumes reduce alveolar ventilation resulting in respiratory acidosis, which can be mitigated through the application of extracorporeal carbon dioxide removal (ECCO_2_R) [7–9].

The feasibility and safety of ultra-protective ventilation strategies facilitated by ECCO_2_R has been tested in several studies using a pump-less arteriovenous device operating at a blood flow rate of 1.0–1.5 L/min [10–12]. Information on feasibility and safety of ultra-protective ventilation strategies facilitated by low-flow venous-venous ECCO_2_R are limited to a single-center study [8].

The aim of the current study was to assess in a multicenter trial the feasibility and safety of an ultra-protective ventilation strategy facilitated by low-flow veno-venous ECCO_2_R in patients with moderate ARDS. We used an ECCO_2_R system (Hemolung Respiratory Assist System, ALung Technologies), which is specifically designed to provide clinically significant CO_2_ removal at low blood flow rates (350–550 mL/min).

## Methods

Patients were enrolled in four European intensive care units of academic hospitals. Local ethics committees approved the study protocol. Informed consent was obtained from the patients. In the case of incompetent patients, consent was obtained in accordance with local ethics committee procedures [13].

### Patients

The study included fifteen adult patients with moderate ARDS according to the Berlin definition (PaO_2_/FiO_2_ (P/F) 100–200 mmHg, with positive end-expiratory pressure (PEEP) >5 cmH_2_O) [14], who were mechanically ventilated with an expected duration of ventilation longer than 24 h. Exclusion criteria were age <18 years, pregnancy, decompensated heart insufficiency or acute coronary syndrome, severe chronic obstructive pulmonary disease (COPD), respiratory acidosis with arterial PCO_2_ (PaCO2) >80 mmHg, acute brain injury, severe liver insufficiency (Child-Pugh score >7) or fulminant hepatic failure, heparin-induced thrombocytopenia, contraindication for systemic anticoagulation, patient moribund, decision to limit therapeutic interventions, catheter access to femoral vein or jugular vein impossible, pneumothorax, or platelet count <50 × 10^3^/mL.

### ECCO_2_R System

Low-flow ECCO_2_R was provided with the Hemolung Respiratory Assist System (RAS) (ALung Technologies, Inc, Pittsburgh, PA, USA) [15]. Briefly, venous blood is circulated through a 15.5-Fr dual lumen venous catheter (jugular or femoral) by a magnetically driven centrifugal pump at a flow rate of 350–550 mL/min. The pump is integrated within a cylindrical bundle of hollow fiber membranes, creating a flow pattern, which improves CO_2_ transfer efficiency relative to passive oxygenators. Sweep gas (air or 100 % O_2_) is drawn through the hollow fibers under negative pressure by a vacuum pump, creating a gradient for CO_2_ diffusion. Maintaining the sweep gas under negative pressure mitigates the risk of air embolism across the membrane, and also allows for automatic removal of plasmatic water condensation from the fiber lumens in order to preserve gas exchange efficiency. Level of blood flow, pump speed (RPM) and extracorporeal CO_2_ removal rate (vCO_2_) are displayed on a controller.

### Study protocol

Patients were sedated, paralyzed and ventilated in accordance with the EXPRESS trial protocol [16]: V_T_ of 6 mL/kg (ideal body weight); PEEP set to achieve P_plat_ of 28–30 cmH_2_O; respiratory rate (RR) set to 20–35 to maintain approximately the same minute ventilation as before study initiation. Percutaneous venous femoral or jugular cannulation was performed through insertion of a single dual-lumen catheter (15.5 Fr) after administration of a heparin bolus (80 IU/kg). The device was activated at a blood flow rate of 350–550 mL/min and a sweep gas of 0 L/min such that no CO_2_ removal was initially performed.

Following a 2-h run-in time, V_T_ was gradually reduced from 6 to a minimum value of 4 mL/kg by 0.5 mL/kg every 30 minutes and PEEP was increased to target a P_plat_ between 23 and 25 cmH_2_O . If arterial pH was <7.25 with PaCO_2_ >60 mmHg, despite an increase in RR up to 35/min, sweep gas through the ECCO_2_R device was switched on with 100 % oxygen at 10 L/min to obtain an arterial pH ≥7.25 with a PaCO_2_ ≤60 mmHg and RR ≤35/min. If PaCO_2_ was >75 mmHg and/or pH <7.20, despite a respiratory rate of 35/min and optimized ECCO_2_R, sodium bicarbonate could be infused. If undesirable hypercapnia/acidosis persisted, V_t_ was increased at the discretion of the treating physician. Refractory hypoxemia and/or hypercapnia could be managed at the discretion of the attending physician using veno-venous extracorporeal membrane oxygenation (ECMO), prone positioning, or nitric oxide (NO) inhalation. If PaCO_2_ was constantly <35 mmHg and/or pH was >7.50 under the aforementioned ECCO_2_R settings, the respiratory rate was decreased to 18–22/min and sweep gas flow was decreased to 2–5 L/min.

The ECCO_2_R-facilitated ultra-protective ventilation strategy was continued for at least 24 h. The potential for weaning from ultra-protective ventilation and ECCO_2_R was assessed daily if PaO_2_/FiO_2_ (P/F) was >200 by setting mechanical ventilation according to conventional ARDSnet settings (V_T_ = 6 mL/kg, PEEP 5–10 cmH_2_O, RR 20–30/min, inspired O_2_ fraction (FiO_2_) = 40 %) and switching off sweep gas through the ECCO_2_R device. Under these conditions, if the patient remained stable for at least 12 h with P_plat_ <25 cmH_2_O and PaCO_2_ <50 mmHg (allowing for RR up to 30–35/min), ECCO_2_R was discontinued and the venous catheter removed.

ECCO_2_R parameters (blood flow, sweep gas flow, and CO_2_ removal rate), ventilator settings (V_T_, PEEP, RR, P_plat_, mean airway pressure, minute ventilation, inspiratory-to-expiratory ratio, inspired fraction of oxygen), hemodynamics (mean arterial pressure, heart rate, dose of vasopressor) and arterial blood gas values (pH, PaO_2_, PaCO_2,_ HCO_3_^–^, lactate), heparin dose and activated partial thromboplastin time ratio (aPTTr) were collected at baseline, after run-in time, 30 minutes after every V_T_ reduction and at least twice a day (08:00 am ± 2 h and 08:00 pm ± 2 h) in the subsequent days on ECCO_2_R. Blood chemistry data were collected daily. Respiratory system compliance [17] and driving pressure [18] were calculated according to the standard formula.

Serious adverse events (SAE) were prospectively defined as: (a) any event that is fatal or immediately life threatening, permanently disabling, severely incapacitating or requires prolonged hospitalization; or (b) any event that may jeopardize the patient and requires medical or surgical intervention to prevent one of the outcomes listed above; and (c) which the attending physician perceives might be directly related to enrollment in the clinical trial. Adverse events were considered to be study-related if the event followed a reasonable sequence from a study procedure and could readily have been produced by the study procedure. Adverse events were considered non study-related if they were related primarily to the underlying disease or to ARDS and its sequelae. Other adverse events not fulfilling the above definition were recorded in the patients’ case report forms (CRFs). Following discontinuation of ECCO_2_R, subjects were monitored for adverse events until hospital discharge or day 8 after enrollment, whichever occurred first.

Data are expressed as mean ± SD. Statistical analysis was performed by one-way analysis of variance for repeated measures, followed by Bonferroni post-hoc test for comparison between different time points (Stata Corp, College Station, TX, USA). *P* <0.05 was considered statistically significant.

## Results

Fifteen patients with moderate ARDS were included in the period April to November 2014. Baseline characteristics of patients enrolled in the study are shown in Table 1.

**Table 1:** Baseline characteristics of patients

Variables	Patients (n = 15)
Age (years)	55 ± 19
Gender (male/female), number	11/4
Body mass index (kg/m^2^)	24 ± 8
Lung injury score (Murray)	3 ± 0.9
Risk factors for acute respiratory distress syndrome, number of patients	
Pulmonary	
Pneumonia	12
Non-pulmonary	
Sepsis	3
Comorbidities, number of patients	
Diabetes mellitus	3
Chronic obstructive pulmonary disease	1
Arterial hypertension	5
Coronary artery disease	1
Chronic renal impairment	2
Atrial fibrillation	2
Alcohol use disorder	4
Obesity	2
Simplified acute physiology score II	51 ± 14
Sequential organ failure assessment	10 ± 4

**Fig. 1:**
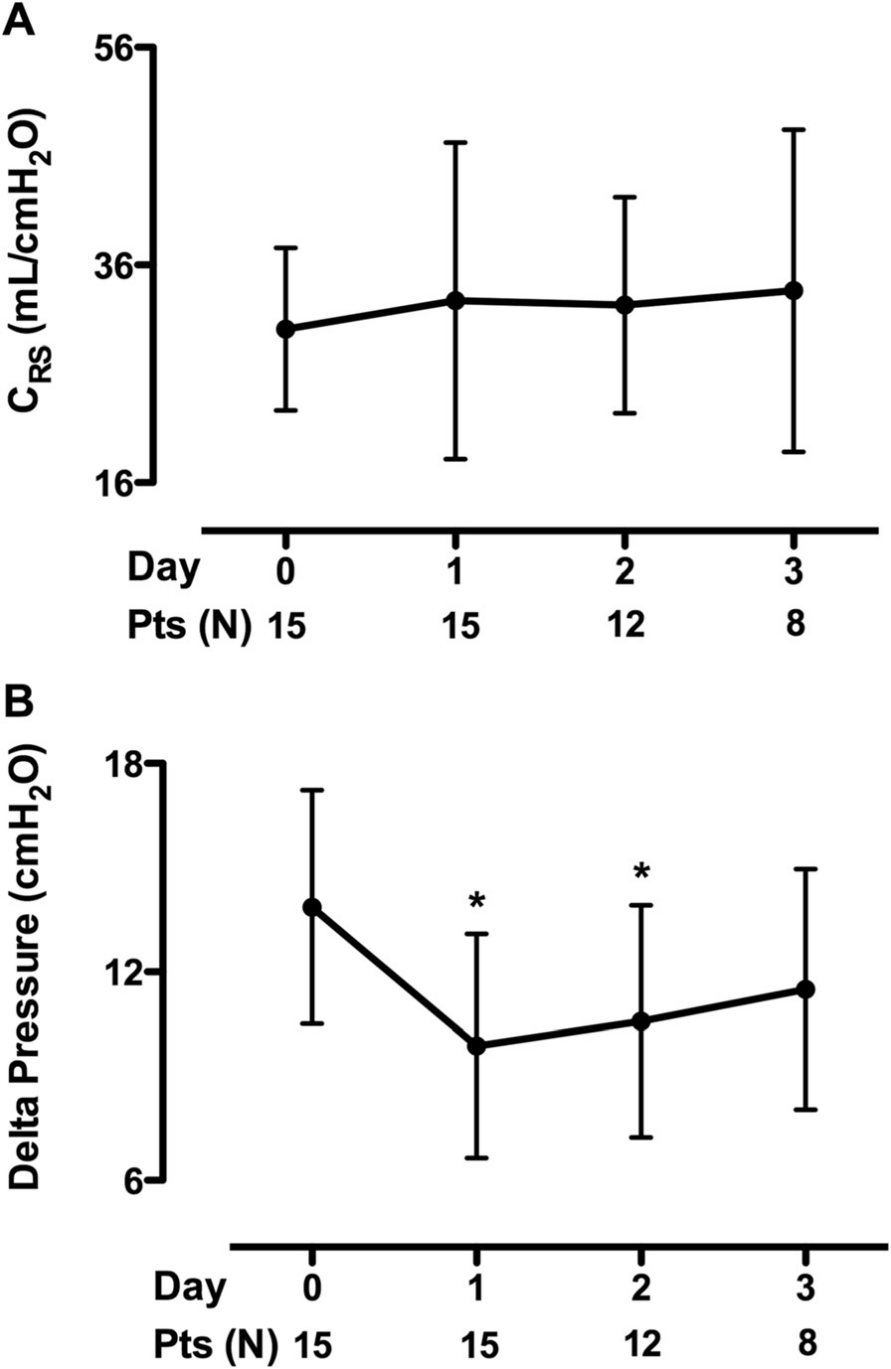
Time course of respiratory system compliance (C_RS_) (**a**) and driving pressure (**b**). *P <0.05 vs day 0. *Pts* patients

Ventilation settings during the V_T_ reduction phase are shown in Table 2. At baseline, all patients had PaO_2_/FiO_2_ ≤200, they were ventilated with a conventional protective ventilation strategy according to the EXPRESS trial protocol, and were paralyzed for a median time of 1 day kk. The initial stepwise reduction in V_T_, without ECCO_2_R, resulted in significant respiratory acidosis (pH <7.25) in all 15 patients at a mean V_T_ of 3.96 ± 0.1 mL/kg. After initiation of ECCO_2_R, a V_T_ of 4.29 ± 0.5 mL/kg was achieved and respiratory acidosis was significantly corrected, with pH and PaCO_2_ returning to within 10 % of baseline values obtained at V_T_ = 6 mL/kg. The median number of days on ECCO_2_R was 3 (2–4). The reduction in V_T_ was associated with a significant reduction in P_plat_ from 27.7 ± 1.6 to 23.9 ± 1 cmH_2_O (*p* <0.05) at day 1 and this difference remained significant throughout the study period (Table 3). PEEP tended to increase from 12 ± 3 to 14 ± 2 cmH_2_O at day 1, however, this difference was not statistically significant over time (Table 3). Driving pressure (P_plat_ – PEEP) was significantly reduced during the first 2 days compared to baseline (*p* <0.05); there were no significant differences in the values of respiratory system compliance (Fig. 1). At day 1, the ECCO_2_R device provided CO_2_ removal of 81 ± 9 mL/min at a blood flow rate of 435 ± 60 mL/min and sweep gas flow rate of 10 ± 0.3 L/min. The efficiency of the ECCO2R system was stable throughout the study period (Table 3). Infusion of bicarbonate and renal replacement therapies for acute kidney injury were never used in this cohort of patients.

**Table 2:** Time course of ventilation variables during the run-in phase

Variables	Baseline	V_T_ 5 mL/kg	V_T_ 4.5 mL/kg	V_T_ 4 mL/kg
V_T_ (mL/kg)	6.2 ± 0.7	5.02 ± 0.1*	4.48 ± 0.1*	3.96 ± 0.1*
Respiratory rate (beats/minute)	28 ± 7	29 ± 4	30 ± 4*	30 ± 5*
Positive end-expiratory pressure (cmH_2_O)	12 ± 3	13.8 ± 3	13.6 ± 4	13.0 ± 4.0
Plateau pressure (cmH_2_O)	27.7 ± 1.6	25.2 ± 1.6*	23.6 ± 1.3*	22.7 ± 1.8*
Patients who reached the pH threshold for ECCO_2_R, n		0	2	15

*P <0.05 vs baseline. *V*_*T*_ tidal volume, *ECCO*_*2*_ *R* extracorporeal carbon dioxide removal

**Table 3:** Time course of ventilation variables, blood gases, ECCO_2_R operational characteristics and hemodynamics at V_T_ 4 mL/kg plus ECCO_2_R

Variables	Baseline		V_T_ 4 mL/kg plus ECCO_2_R			
		Day 1	Day 2		Day 3	
Time (h)	8.00	8.00	8.00	20.00	8.00	20.00
Patients (number)	15	15	10	10	8	6
V_T_ (mL/kg)	6.2 ± 0.7	4.29 ± 0.5*	4.58 ± 0.7*	4.59 ± 0.8*	4.8 ± 0.7*	4.8 ± 0.7*
Respiratory rate (beats/minute)	28 ± 7	31.6 ± 4.6*	29.6 ± 6.8	29.6 ± 6.8	28 ± 7	27.4 ± 8.6
Positive end-expiratory pressure (cmH_2_O)	12 ± 3	14 ± 2	13 ± 3	12 ± 4	13 ± 5	13 ± 3
Arterial partial pressure of oxygen/inspired oxygen fraction	159 ± 34	175 ± 45	185 ± 91	190 57	176 ± 59	176 ± 80
Plateau pressure (cmH_2_O)	27.7 ± 1.6	23.9 ± 1*	24 ± 4*	24 ± 3*	24 ± 3*	23 ± 3*
Blood flow (ml/min)		435 ± 60	424 ± 63	423 ± 35	424 ± 29	436 ± 39
Rotations per minute (RPM)		1407 ± 26	1408 ± 30	1409 ± 32	1411 ± 36	1414 ± 41
Sweep gas (L/min)		8.6 ± 3.5	9.2 ± 2.9	9 ± 3	9.9 ± 0.3	8.7 ± 3.2
CO_2_ removal (ml/min)		81 ± 9	70 ± 29	70 ± 31	81 ± 22	71 ± 11
Ph	7.36 ± 0.1	7.33 ± 0.1	7.39 ± 0.1	7.36 ± 0.1	7.33 ± 0.1	7.4 ± 0.1
PaO2 (mmHg)	95 ± 29	90 ± 22	91 ± 26	84 ± 10	81 ± 15	99 ± 29
PaCO2 (mmHg)	51 ± 15	53 ± 15	51 ± 18	52 ± 17	55 ± 20	49 ± 11
HCO3 (mmol/L)	28 ± 5	27.6 ± 6.1	28.7 ± 6.2	28. ± 6.9	28.1 ± 7.4	28.3 ± 6.03
Lactate (mmol/L)	2 ± 1	2.9 ± 4.8	1.9 ± 1.7	1.8 ± 1.8	1.9 ± 2.0	1.3 ± 0.4
Heart rate (beats/minute)	76 ± 9	90 ± 17	86 ± 15	95 ± 21	94 ± 19	94 ± 17
Mean arterial pressure (mmHg)	98 ± 20	74 ± 14	80 ± 10	76 ± 12	76 ± 19	85 ± 14
Norepinephrine dose (mcg/kg/min)	0.51 ± 0.6	0.45 ± 0.4	0.34 ± 0.3	0.29 ± 0.27	0.57 ± 0.4	0.5 ± 0.6

*P <0.05 vs baseline. *V*_*T*_ tidal volume, *ECCO*_*2*_ *R* extracorporeal carbon dioxide removal

At day one, the heparin dosage was 341 ± 138 IU/kg/day with an aPTT ratio of 1.77 ± 0.7, and remained stable over time. The average baseline value of hemoglobin (Hb) was 10.4 ± 2 gr/dL, and the median Hb threshold for transfusion was 6.9 gr/dL (6.9–7.0). On day 1, four patients received 2.25 ± 0.5 red blood cell (RBC) units and 1.25 ± 2.5 pools of platelets. On day 2, only two patients received 1.5 ± 0.7 units of RBCs. On day 3, only one patient received two units of RBCs.

Two study-related adverse events were observed. In one patient, intravascular hemolysis (plasma-free hemoglobin 401.4 mg/dL) was observed resulting in a discontinuation of ECCO_2_R after 2 days. Kinking of the ECCO_2_R catheter caused a reduction in circuit blood flow in another patient. Individual data for patients who needed rescue therapies for worsening hypoxemia are given in Table 4. The overall mortality at day 28 was 47 %. Among the eight survivors, six were successfully weaned from both ECCO_2_R and mechanical ventilation; while two patients were still dependent on ventilator support at 28 days.

**Table 4:** Oxygenation and outcomes of patients who required rescue therapies

Patients	Time of PaO_2_/FiO_2_ worsening	PaO_2_/FiO_2_ before rescue therapy		Outcome (at 28 days)
	(day)	Prone position	ECMO	
# 1	5	108		Dead
# 5	6		78	Alive
# 8	2		75	Dead
# 11	2	115		Alive
# 12	1	158		Dead
# 13	1	162		Alive

*PaO*_*2*_ */FiO*_*2*_ arterial partial pressure of oxygen/inspired oxygen fraction, *ECMO* extracorporeal membrane oxygenation

## Discussion

The main finding of the current study is that the low-flow ECCO_2_R system effectively prevents respiratory acidosis consequent to the reduction of tidal volume to 4 mL/kg in patients with moderate ARDS. Severe hypoxemia occurred in about one third of the patients and was managed by prone positioning in conjunction with ECCO_2_R; conversion to VV-ECMO was required in two patients. Side effects related to ECCO_2_R (intravascular hemolysis and pump malfunction by femoral catheter kinking) were observed in two patients.

The landmark study by the ARDSnet demonstrated a 9 % mortality reduction in patients with ARDS by limiting plateau pressure to <30 cmH_2_O [1]. However, recent studies have shown that patients with ARDS may still be at risk of VILI despite values of P_plat_ ≤30 cmH_2_O [2, 4, 5]. Terragni [2] and Bellani [4] showed that in some patients, tidal hyperinflation may still occur despite limiting V_T_ to 6 mL/kg and P_plat_ to 30 cmH_2_O, and that this is associated with biological signs of VILI, such as higher levels of inflammatory mediators and increased metabolic activity of the lungs. Grasso and coworkers showed that hyperinflation might be attenuated by reducing the levels of PEEP based on the shape of the airway pressure time curve and that this is associated with lower levels of pulmonary inflammatory cytokine release [5]. Moreover, Hager and coworkers found that ARDS patients may benefit from V_T_ reduction even if they already have P_plat_ <30 cmH_2_O [6].

ECCO_2_R has been proposed to partially clear CO_2_ and consequently reduce the need of minute ventilation as delivered by conventional mechanical ventilation [9]. The first evidence that ECCO_2_R might be a safe adjunctive therapy to conventional mechanical ventilation for ARDS patients dates back to the early 1980s [19, 20]. At that time, a modified veno-venous ECMO circuit with a blood flow not >1 L/min allowed for dramatic reductions of minute ventilation and ventilator-applied pressure [19]. More recently, Terragni and colleagues demonstrated that a modified renal replacement therapy circuit with an oxygenator in series with the hemofilter could facilitate an ultra-protective ventilation strategy, which may mitigate VILI [8]. Our results extend these findings by performing a prospective multicenter study in 15 ARDS patients across four European ICUs.

Monitoring of respiratory mechanics may help clinicians in assessing the risk of VILI [17]. In the presence of normal chest wall elastance, values of P_plat_ around 30 cmH_2_O may increase the risk of alveolar hyperinflation, which is the main determinant of VILI [2]; analysis of a large dataset including ARDS patients previously enrolled in clinical trials, shows that driving pressure (i.e., the ratio of tidal volume to respiratory system compliance) is an independent risk factor for hospital mortality, because regardless of the changes in V_T_ and PEEP, only changes in driving pressure affected the outcome [18]. Our data show that an ultra-protective ventilation strategy facilitated by low-flow veno-venous ECCO_2_R resulted in values of respiratory mechanics associated with enhanced protection from VILI as: (1) values of P_plat_ around 25 cmH_2_O, as achieved in this study, have been associated with lower serum levels of the pro-inflammatory cytokine interleukin-6 and a smaller percentage of lung hyperinflation, as demonstrated by computed tomography [2, 7, 8]; and (2) our ultra-protective ventilation strategy resulted in a significant reduction in driving pressure. These findings allow us to speculate that in our patients, global interaction between moderate-to-high levels of PEEP and very low tidal volume, facilitated by ECCO_2_R, might be beneficial to minimize the risk of VILI.

In recent years, advances in technology have generated renewed interest in all extracorporeal support techniques. While high flow veno-venous ECMO has been increasingly used to treat life-threatening hypoxemia [21, 22], ECCO_2_R systems are used to provide partial-to-full CO_2_ removal with minimal effects on oxygenation [9]. Appropriate strategies to manage worsening hypoxemia during ECCO_2_R treatment is a compelling issue. In fact, depending on the severity of lung injury, patients with ARDS may experience worsening of arterial oxygenation that might be life threatening [23]. Moreover, the use of very low tidal volumes may expose patients to the risk of de-recruitment in the event that insufficient PEEP is applied. Consequently, prone positioning while the patient is still on ECCO_2_R, or shift to high-flow veno-venous ECMO, might be necessary. In this regard, prone positioning has been demonstrated to be effective not only in improving oxygenation but also in decreasing early and late mortality [24]. In the current study, indication for rescue therapies for profound hypoxemia was not established by protocol. Within the first week of enrollment 27 % of patients in our cohort required prone positioning when their PaO2/FiO2 dropped to a median value of 137. Notably, patients underwent prone position without any interruption of ECCO_2_R and had improvement in arterial oxygenation. Only two patients required escalation from ECCO_2_R to ECMO because of life-threatening hypoxemia. Mortality at 28 days was 47 %, which was expected in a cohort of patients with moderate and severe ARDS.

Previous-generation ECCO_2_R systems have carried a high rate of mechanical complications such as pump malfunction, membrane clotting, and catheter displacement [8]. In the current study, target blood flow rates were not reached in one case only due to a kinked catheter; otherwise, the treatment was consistent over time. Compared with arterio-venous systems in which limb ischemia, compartment syndrome, and intracranial hemorrhage have been described [7], only one case of intravascular hemolysis requiring transfusion was reported in our series.

Some limitations of this study should be addressed. First, inferences from this study are limited by its small sample size. Second, although we speculate that the strategy tested in this multicenter study was lung-protective, we did not measure pro-inflammatory mediators associated with VILI and we did not evaluate lung volume/densities distribution with computed tomography of the chest. The precise impact of worsening oxygenation during ECCO_2_R treatment is also not clear. Consequently, this approach will be systematically tested in an upcoming randomized clinical trial, such as that under the auspices of European Society of Intensive Care Medicine, which will test the feasibility, safety and efficacy of several ECCO_2_R systems to facilitate ultra-protective ventilation - V_T_ of 4 mL/kg predicted body weight (PBW) and P_plat_ <25 cmH_2_O - in patients with moderate and severe ARDS (SUPERNOVA: a strategy of ultraprotective lung ventilation with extracorporeal CO2 removal for new-onset moderate to severe ARDS) [25].

## Conclusions

In conclusion, low-flow ECCO_2_R is feasible, safe and efficient. It facilitates an ultra-protective mechanical ventilation strategy for reducing tidal volume to 4 mL/kg to maintain P_plat_ <25 cmH_2_O. This approach allows the delivery of mechanical ventilation with relatively low driving airway pressures while preserving sufficient gas exchange and preventing hypercapnia and respiratory acidosis due to reduced ventilation. The current study provides a clinical and physiological rationale for testing whether ultra-protective mechanical ventilation improves clinical outcomes of patients with moderate and severe ARDS in randomized control trials.

## Key messages

- Low-flow extracorporeal CO_2_ removal safely and effectively facilitates an ultra-protective ventilation strategy in patients with moderate ARDS
- The current study provides a clinical and physiological rationale to study whether ultra-protective mechanical ventilation improves clinical outcomes of patients with moderate and severe ARDS in randomized control trials
